# Effects of Chronic Physical Exercise or Multicomponent Exercise Programs on the Mental Health and Cognition of Older Adults Living in a Nursing Home: A Systematic Review of Studies From the Past 10 Years

**DOI:** 10.3389/fpsyg.2022.888851

**Published:** 2022-05-13

**Authors:** Jason Leonardo Da Silva, Nounagnon Frutueux Agbangla, Christine Le Page, Wahiba Ghernout, Bernard Andrieu

**Affiliations:** ^1^Institut des Sciences du Sport-Santé de Paris (URP 3625), Université Paris Cité, Paris, France; ^2^Maison de Retraite Villa Jules Janin, Paris, France; ^3^Laboratory URePSSS – SHERPAS (ULR 7369), Univ. Artois, Univ. Littoral Côte d'Opale, Univ. Lille, UFR STAPS, Liévin, France

**Keywords:** aging, nursing homes, mental health, cognitive functions, physical exercise

## Abstract

Some nursing homes for the elderly provide holistic care that integrates several interventions, including physical exercise. The aim of this systematic review is to summarize the effects of physical exercise or multicomponent exercise programs on the mental health (wellbeing, anxiety and depression) and cognitive functions of older adults with/without dementia who live in a nursing home and do/do not require wheelchair assistance. To this end, PubMed, PsycInfo and Web of Science are using to identify clinical trials and randomized controlled studies conducted during the period January 2011 to December 2021 to examine the progression of research in this field over the past ten years. In total, 2597 articles are identifying and 21 are including in the systematic review. After selecting articles according to the PRISMA standards, the data extraction and methodological quality assessment of the eligible studies are performing individually by two reviewers and then pooled together. The synthesis of the studies shows that physical exercise or multicomponent exercise programs have a beneficial effect on mental health and cognitive functions. However, this effect is more common among older adults without dementia, compared to their counterparts who have dementia or use wheelchairs. The explanatory mechanisms, lack of benefits from physical exercise and the need to standardize methods are discussing in this regard. Finally, future studies must examine the effects of an innovative and adaptive physical activity program on the mental health and cognitive functions of older adults living in nursing homes.

## Introduction

Since the 1990s, the proportion of individuals over the age of 60 has continued to increase; according to projections, it will reach 21.1% of the world population by 2050 (Sander et al., 2015). But this increase in the proportion of older adults in recent decades, which reflects the increasing life expectancy, does not necessarily reflect better health for the elderly (Valenzuela et al., 2019). Indeed, while some older adults retain good physical and cognitive abilities, which allow them to have a strong social commitment (Rowe and Kahn, 1997; Le Deun and Gentric, 2007), others develop pathologies (depression, dementia, locomotion disorders, sensory disorders and cardiorespiratory conditions) (Le Deun and Gentric, 2007), which can lead to disabilities. The prevalence of these conditions exacerbates after the age of 85 (Guralnik et al., 1993; Cordes et al., 2019). Thus, the increase in the number of people aged over 60 is also accompanied by the increase in the number of older adults with disabilities that affect their daily activities. To provide a better standard of living for these dependent adults, they have to be institutionalized in nursing homes.

The care given in nursing homes is both specific and holistic, depending on whether the older adults have physical or cognitive disabilities. However, even if nursing homes offer solutions to these vulnerable older adults, they still exhibit cognitive and functional decline (González-Colaço Harmand et al., 2014; Jerez-Roig et al., 2017). To counteract this, physical exercise—a subcategory of physical activity that is planned, structured and repetitive and promotes the maintenance or development of physical fitness (Thivel et al., 2018),—is an intervention provided to the residents. This choice is justified by the fact that many studies have shown the benefits of physical exercise on the cognition and/or physical abilities of healthy older adults (for review, see Kramer and Colcombe, 2018; Falck et al., 2019) or older adults residing in care homes (Aguirre and Villareal, 2015; Brett et al., 2016; Kocic et al., 2018; Baldelli et al., 2021; Li et al., 2021). In older adults living in care homes with or without illnesses, the latest systematic reviews (Brett et al., 2016; Baldelli et al., 2021) and meta-analyses (Li et al., 2021) focused on the overall effects of physical exercise on psychological factors, showing the positive impact on quality of life, depression and wellbeing. However, the two systematic reviews, notwithstanding their qualities, focused on different populations and psychological factors. For example, in the systematic review by Baldelli et al. (2021), the included studies investigated the effect of physical exercise on the quality of life of healthy older adults. However, in the systematic review by Brett et al., the studies highlighted the effects of physical exercise on the wellbeing of patients suffering from dementia (Brett et al., 2016). Thus, no systematic review has highlighted or analyzed the research that investigated the effects of physical exercise on mental health (depression, anxiety and wellbeing) and cognition (overall cognitive functioning, information processing speed and executive functions) among older adults living in nursing homes with or without illnesses.

The objective of this systematic review is to summarize the studies that have examined the effect of physical activity or multicomponent training on the mental health and cognitive functions of older adults with/without dementia who live in a nursing home and do/do not require wheelchair assistance. It will look at studies from the past 10 years.

## Methods

### Data Search Strategy

To perform this systematic review, a comprehensive literature search was conducted on the PubMed, Web of Science and PsycInfo databases between January 1, 2011 and December 22, 2021 to identify the relevant studies. We have chosen this period to examine the progression of research in this field over the past 10 years. During this search process, the following keywords matches were used: “exercise” OR “physical activity” AND “elderly nursing home residents” WITH “well-being”; “exercise” OR “physical activity” AND “elderly nursing home residents” WITH “executive functioning” OR “inhibition” OR “working memory updating” OR “cognitive flexibility”; “exercise” OR “physical activity” AND “elderly nursing home residents” WITH “anxiety”; “exercise” OR “physical activity” AND “elderly nursing home residents” WITH “depression.” Moreover, the filters “Randomized Controlled Trial” and “Clinical Trial” were also activated.

### Study Selection

**Figure 1:**
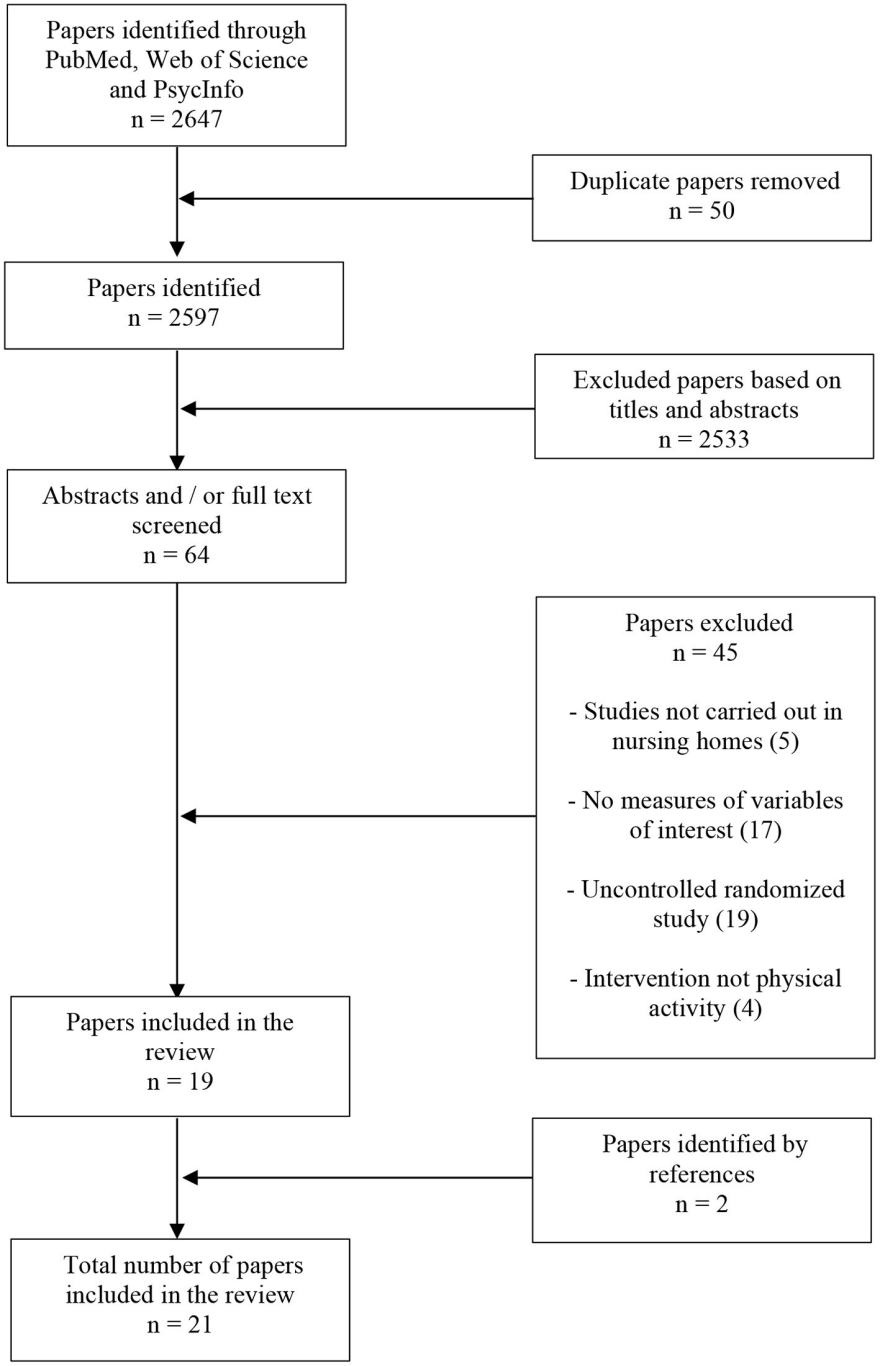
Chart flow of the selection process.

After the identification step, we proceeded to the selection step which involves screening titles and abstracts to remove duplicate and irrelevant studies. Then, the full text of the eligible studies was independently reviewed by two authors (J. L. Da S and N. F. A) to include studies that met the following criteria: (a) The average age of the participants must be ≥65 years; (b) the experimental design must be a randomized controlled trial or a cluster randomized controlled trial; (c) the study must be conducted in nursing homes; (d) the language of the publication must be in English; (e) the study must investigate the effect of an exercise program on at least one of our variables of interest which are depression, anxiety, wellbeing, global cognitive functioning and core executive functions (inhibition, working memory updating and cognitive flexibility). In addition to these inclusion criteria, reviews, meta-analyses and studies that presented only a randomized controlled protocol without results were excluded. At the end of this inclusion work, the two authors synthesized the list of articles to be included and, finally, the references of the recent systematic reviews and meta-analyses identified were scanned to add the relevant articles to the list. The selection procedure is illustrated in Figure 1.

### Data Extraction

For each article included, one author (J. L. Da S) used a standardized extraction form to extract the data. This extraction was then checked by a second author (N. F. A) to make sure there were no errors. The standardized extraction form contained the following variables: the references, characteristics of the participants and tools or tasks used to measure the variables of interest, the intervention and major outcomes.

### Methodological Quality Assessment

The methodological quality of the studies included in our systematic review was assessed by the Jadad scale (Jadad et al., 1996). This scale was used in a systematic review by Baldelli et al. (2021) and includes five items: Item (1) Was the study described as randomized?; Item (2) Was the method used to generate the sequence of randomization described and appropriate?; Item (3) Was the study described as double-blind?; Item (4) Was the method of double-blinding described and appropriate?; Item (5) Was there a description of withdrawals and dropouts? One point is allocated to each item if the answer to the item is “yes.” On the contrary, one point is deducted “if the method to generate the sequence of randomization was described and was inappropriate” or if “the study was described as double blind, but the method of blinding was inappropriate” (Jadad et al., 1996). The total score ranges from 0 to 5 points. All the assessments of the studies are summarized in Table 1.

**Table 1:** Assessment of the methodological quality of the studies.

**References**	**Item 1**	**Item 2**	**Item 3**	**Item 4**	**Item 5**	**Total score**
Arrieta et al. (2020)	1	1	0	0	1	3
Barthalos et al. (2016)	1	0	0	0	1	2
Bischoff et al. (2021)	1	1	0	0	1	3
Chen et al. (2015)	1	1	0	0	1	3
Cheng et al. (2014)	1	1	0	0	1	3
Cordes et al. (2021)	1	1	0	0	1	3
de Souto Barreto et al. (2017)	1	1	0	0	1	3
Fakhari (2017)	1	1	0	0	1	3
Frändin et al. (2016)	1	0	0	0	1	2
Henskens et al. (2018)	1	1	0	0	1	3
Kim and Kang (2021)	1	1	0	0	1	3
Lok et al. (2017)	1	1	0	0	1	3
Moreira et al. (2018)	1	0	1	0	0	2
Rezola-Pardo et al. (2019)	1	1	0	0	1	3
Rezola-Pardo et al. (2020)	1	1	0	0	1	3
Sanders et al. (2020)	1	1	0	0	1	3
Tapps et al. (2013)	1	0	0	0	0	1
Telenius et al. (2015a)	1	1	0	0	1	3
Telenius et al. (2015b)	1	1	0	0	1	3
Tse et al. (2014)	1	1	0	0	1	3
Underwood et al. (2013)	1	1	0	0	1	3

## Results

### Characteristics of the Studies

After removing the duplicate items, we found 2,597. Following this, we scanned the titles to exclude 2,533. The next step was to read the remaining 64 articles fully to identify the eligible studies. We found 19 eligible items, to which we added two items by checking the references. A total of 21 articles (Tapps et al., 2013; Underwood et al., 2013; Cheng et al., 2014; Tse et al., 2014; Chen et al., 2015; Telenius et al., 2015a,b; Barthalos et al., 2016; Frändin et al., 2016; de Souto Barreto et al., 2017; Fakhari, 2017; Lok et al., 2017; Henskens et al., 2018; Moreira et al., 2018; Rezola-Pardo et al., 2019, 2020; Arrieta et al., 2020; Sanders et al., 2020; Bischoff et al., 2021; Cordes et al., 2021; Kim and Kang, 2021) were included in the current systematic review. Of these, 13 were conducted in Europe (Underwood et al., 2013; Telenius et al., 2015a,b; Barthalos et al., 2016; Frändin et al., 2016; de Souto Barreto et al., 2017; Henskens et al., 2018; Rezola-Pardo et al., 2019, 2020; Arrieta et al., 2020; Sanders et al., 2020; Bischoff et al., 2021; Cordes et al., 2021), six in Asia (Cheng et al., 2014; Tse et al., 2014; Chen et al., 2015; Fakhari, 2017; Lok et al., 2017; Kim and Kang, 2021) and two in the America (Tapps et al., 2013; Moreira et al., 2018). Out of these eligible studies, 16 were single randomized controlled trials, while five were cluster randomized controlled trials. Moreover, 15 of these were conducted in multi-center settings.

### Characteristics of the Participants

Some of the studies selected in our review included older adults who did not have dementia and/or did not use wheelchair assistance (Tapps et al., 2013; Underwood et al., 2013; Tse et al., 2014; Barthalos et al., 2016; Frändin et al., 2016; Fakhari, 2017; Lok et al., 2017; Moreira et al., 2018; Rezola-Pardo et al., 2019, 2020; Arrieta et al., 2020; Bischoff et al., 2021; Kim and Kang, 2021). The overall sample size for these studies was 2,124 participants, with 1,060 (females: 71.4%) participants in the intervention groups and 1,064 (females: 74.8%) participants in the control groups. The mean age of the participants was between 69 and 86 years and their body mass index was between 22 and 29 kg/m^2^. Another section of the selected studies involved older adults who did have dementia or use wheelchair assistance (Cheng et al., 2014; Chen et al., 2015; Telenius et al., 2015a,b; de Souto Barreto et al., 2017; Henskens et al., 2018; Sanders et al., 2020; Cordes et al., 2021). In total, 856 participants were included in these studies with 441 (females: 67%) participants in the intervention groups and 415 (females: 73.6%) participants in the control groups. The mean age of these participants was between 79 and 88 years while the body mass index was between 24 and 27 kg/m^2^. Moreover, apart from having dementia or being in a wheelchair, these participants had other pathological conditions, such as high blood pressure, chronic obstructive pulmonary disease, type 2 diabetes and stroke.

### Quality Assessment of the Studies

Following the assessment of these 21 studies using the Jadad scale, it was seen that 17 studies have a score of three, while four have a score of <3. This result reflects that most studies are of good quality. The overall results of the evaluation are summarized in Table 1.

### Measures of Mental Health and Cognitive Functions

Mental health is defined as “a state of the successful performance of mental functions, resulting in productive activities, fulfilling relationships with people and the ability to adapt and to cope with adversity” (Bidonde et al., 2014). Based on this definition, we can see that mental health is influenced by several factors, including (needless to say) psychological factors. For our systematic review, we chose depression, anxiety and wellbeing as psychological factors that can reflect mental health. We found that the studies included in this review used different tools to measure wellbeing: short form of the Health Survey (Tse et al., 2014; Bischoff et al., 2021; Cordes et al., 2021); 36-Item Short Form Survey (Lok et al., 2017); Satisfaction with Life Scale (Bischoff et al., 2021); The World Health Organization Quality of Life questionnaire (Barthalos et al., 2016); European Quality of Life-5 (Underwood et al., 2013); Quality of life in Alzheimer's Disease scale (Rezola-Pardo et al., 2019, 2020; Arrieta et al., 2020); Quality of Life in Late-Stage Dementia scale (Telenius et al., 2015a,b); The Philadelphia Geriatric Center Morale Scale (Frändin et al., 2016); Goldberg Anxiety and Depression Scale (Rezola-Pardo et al., 2019, 2020; Arrieta et al., 2020); Beck's Depression Inventory (Tapps et al., 2013; Fakhari, 2017; Lok et al., 2017); Geriatric Depression Scale (Underwood et al., 2013; Cheng et al., 2014; Tse et al., 2014; Kim and Kang, 2021); Cornell Scale for Depression (Telenius et al., 2015a,b; Henskens et al., 2018); Center for Epidemiological Studies Depression scale (Cordes et al., 2021); Taiwanese Depression Questionnaire (Chen et al., 2015); Geriatric Anxiety Inventory (Kim and Kang, 2021).

Cognitive functions are mental processes that allow us to process the information we receive from the environment and respond appropriately. In the selected studies, several tasks or questionnaires have been used to measure cognitive functions such as global cognitive functioning (Mini-Mental State Examination) (Underwood et al., 2013; Cheng et al., 2014; Telenius et al., 2015a,b; Frändin et al., 2016; de Souto Barreto et al., 2017; Henskens et al., 2018; Sanders et al., 2020; Kim and Kang, 2021); Montreal Cognitive Assessment (Moreira et al., 2018; Rezola-Pardo et al., 2019, 2020; Arrieta et al., 2020; Cordes et al., 2021); short form of the Severe Impairment Battery (Henskens et al., 2018), speed of information processing [Symbol search and coding tests from the Wechsler Adult Intelligence Scale (Arrieta et al., 2020)], psychomotor speed [Trail Making Test A (Rezola-Pardo et al., 2019; Arrieta et al., 2020; Sanders et al., 2020)], memory [Rey Auditory Verbal Learning Test (Rezola-Pardo et al., 2019, 2020); digit span forward and backward, 15-word immediate and 30-min delayed recall, impaired word-list delayed recall (Cheng et al., 2014; Henskens et al., 2018); serial subtraction test (Cordes et al., 2021); fluency task (Cheng et al., 2014; Henskens et al., 2018; Rezola-Pardo et al., 2019; Arrieta et al., 2020; Sanders et al., 2020)] and executive functions [go/no go test (Henskens et al., 2018); Stroop test (Sanders et al., 2020)].

### Characteristics of the Interventions

The interventions used in the experimental and control groups have been summarized in Supplementary Tables 1, 2 according to whether the participants are dementia-free or wheelchair-free. In this section, we will focus on the interventions of the experimental groups. Indeed, most studies that included older adults without dementia and without wheelchairs used multicomponent interventions (Underwood et al., 2013; Tse et al., 2014; Barthalos et al., 2016; Frändin et al., 2016; Moreira et al., 2018; Rezola-Pardo et al., 2019, 2020; Arrieta et al., 2020; Bischoff et al., 2021; Kim and Kang, 2021). These multicomponent interventions involve either several components of physical activity (strength + balance + walking + stretching) or some components of physical activity and cognitive exercises or regular activity therapy or depression awareness training. The interventions usually lasted between 8 weeks and 12 months with a frequency of two to three sessions per week. Each session had a duration of 45–60 min with a moderate intensity. In addition to these interventions, other studies have proposed Tai Chi (Fakhari, 2017), walking (Lok et al., 2017) or resistance training (Tapps et al., 2013). These interventions lasted 10–12 weeks with a frequency of three to four sessions per week. Each session was of 20–30 min with low to moderate intensity.

The studies carried out among older adults with dementia or older adults who need wheelchair assistance have also mostly proposed multicomponent interventions (Chen et al., 2015; Telenius et al., 2015a,b; de Souto Barreto et al., 2017; Henskens et al., 2018; Sanders et al., 2020; Cordes et al., 2021). These interventions are the same as those of the studies which included seniors without dementia or wheelchairs (regular daily + wheelchair-bound senior elastic band/Coordination + motor cognitive games, aerobic, stretching/coordination + balance + strengthening + aerobic). They lasted from 16 weeks to 6 months with a frequency of two to three sessions per week. Each session was of 40–60 min with a low to high intensity. Out of these, only one study proposed an intervention that was not multicomponent. This intervention comprised practicing Tai Chi for 12 weeks (three times per week, an hour per session).

### Effects of the Interventions on Mental Health and Cognitive Functions

In older adults without dementia who do not need wheelchair assistance, some studies showed the interventions alleviating conditions such as depression (Tapps et al., 2013; Tse et al., 2014; Fakhari, 2017; Lok et al., 2017; Kim and Kang, 2021) and anxiety (Rezola-Pardo et al., 2019, 2020; Kim and Kang, 2021). However, other studies failed to show the same (Underwood et al., 2013; Arrieta et al., 2020).

Regarding wellbeing, the results were mostly inconsistent. Indeed, while some studies observed the intervention's effect on wellbeing when measured with different quality of life questionnaires (Tse et al., 2014; Lok et al., 2017; Rezola-Pardo et al., 2019, 2020; Kim and Kang, 2021), others were unable to demonstrate the same (Underwood et al., 2013; Barthalos et al., 2016; Frändin et al., 2016; Arrieta et al., 2020; Bischoff et al., 2021). As for cognitive functions, the results of the studies globally suggest that the elderly improve their global cognitive functioning (Moreira et al., 2018; Arrieta et al., 2020; Kim and Kang, 2021) or maintain it (Rezola-Pardo et al., 2019, 2020) following the interventions. Moreover, the interventions are also beneficial for the older adults' processing speed (Arrieta et al., 2020) and help to maintain their memory (Rezola-Pardo et al., 2019, 2020). However, the study by Frändin et al. (2016) was unable to demonstrate these effects. All studies are presented in detail in Supplementary Table 1.

For studies that had interventions with a strong physical component for depressed older adults with dementia or depressed older adults who need wheelchair assistance, the results were mixed. Among older adults using wheelchairs, the intervention reduced depressive symptoms (Chen et al., 2015; Cordes et al., 2021). On the other hand, in older adults with dementia, no significant effect was observed (Cheng et al., 2014; Telenius et al., 2015a,b), except for one study wherein the effect was found only among men (Henskens et al., 2018). Similarly, the interventions could not substantially influence the wellbeing of older adults with dementia or older adults who need wheelchair assistance (Telenius et al., 2015a,b; Cordes et al., 2021). Regarding cognitive functions, some studies highlighted the positive effects of the interventions on global cognitive functioning, especially memory and verbal fluency (Cheng et al., 2014; Henskens et al., 2018; Cordes et al., 2021). However, there were some studies that did not observe any effects for the same (Telenius et al., 2015a,b; de Souto Barreto et al., 2017; Sanders et al., 2020). All studies are presented in detail in Supplementary Table 2.

## Discussion

The aim of this systematic review was to synthesize the research conducted over the past 10 years on the effects of physical exercise or multicomponent exercise programs on the mental health and cognitive functions of older adults living in nursing homes. Specifically, we examined if the effect of the interventions depended on whether or not the older adults had dementia or were wheelchair-bound. Most of the studies in our review had a Jadad score of 3, which indicates their good methodological quality (Underwood et al., 2013; Cheng et al., 2014; Tse et al., 2014; Chen et al., 2015; Telenius et al., 2015a,b; de Souto Barreto et al., 2017; Fakhari, 2017; Lok et al., 2017; Henskens et al., 2018; Rezola-Pardo et al., 2019, 2020; Arrieta et al., 2020; Sanders et al., 2020; Bischoff et al., 2021; Cordes et al., 2021; Kim and Kang, 2021). The other four had a Jadad score less than 3 (Tapps et al., 2013; Barthalos et al., 2016; Frändin et al., 2016; Moreira et al., 2018). Although these four studies had a poor methodology, we chose to include them in our review because of the small number of randomized controlled studies based on the topic of our review. The results showed these interventions as having a beneficial effect on mental health (depression, anxiety, and wellbeing) among older adults without dementia or older adults who do not need wheelchair assistance (Tapps et al., 2013; Tse et al., 2014; Fakhari, 2017; Lok et al., 2017; Rezola-Pardo et al., 2019, 2020; Kim and Kang, 2021). In contrast, among the older adults who do have dementia and use wheelchairs, these interventions had no significant effect on their mental health, barring few exceptions (Chen et al., 2015; Henskens et al., 2018; Cordes et al., 2021). For cognitive functions, these interventions were shown capable of improving or maintaining the global cognitive functioning, processing speed or memory among older adults without dementia or older adults who do not need wheelchair assistance (Moreira et al., 2018; Rezola-Pardo et al., 2019, 2020; Arrieta et al., 2020; Kim and Kang, 2021). In contrast, among their counterparts, the results were inconsistent; some studies showed these interventions to be of benefit (Cheng et al., 2014; Henskens et al., 2018; Cordes et al., 2021) while others did not (Telenius et al., 2015a,b; de Souto Barreto et al., 2017; Sanders et al., 2020).

### Explanations for the Beneficial Effects of Physical Exercise or Multicomponent Exercise Programs

The beneficial effects of these interventions on the mental health and cognitive functions of older adults living in nursing homes could be explained by the fact that they improve cognitive vitality (Groot et al., 2016). This can be further explained by several physiological mechanisms (Umegaki et al., 2021). Physical exercise or multicomponent exercise programs can lead to the release of neurotrophic factors, such as a brain-derived neurotrophic factor (Rehfeld et al., 2018; Ruiz-González et al., 2021) and insulin growth factor 1 (Kang et al., 2020; Stein et al., 2021), which can cross the blood-brain barrier to induce the synaptic plasticity and neurogenesis that to improve cognition. Moreover, the increase in the release of a brain-derived neurotrophic factor after physical activity can positively influence depressive symptoms (Penseyres and Martin, 2018). Besides this factor, other secretions such as dopamine and serotonin can explain the reduction in depressive symptoms following physical exercise (Dishman et al., 2006; De Matos et al., 2009). Another physiological mechanism that may explain the improvement in cognitive vitality is the release of the vascular endothelial growth factor which is a precursor to angiogenesis. Angiogenesis induces an increase in cerebral blood flow which, in turn, ensures a better oxygen supply to the neuronal networks involved in cognitive functions so that they can function better (Albinet et al., 2014; Dupuy et al., 2015; Agbangla et al., 2021). Moreover, these interventions are also capable of reducing oxidative stress (Umegaki et al., 2021) and inflammation (Fedewa et al., 2017; Huang et al., 2021) which can reduce neuronal death and consequently maintain the cognitive functions. Apart from these physiological mechanisms, another mechanism, a social one, could also explain the improvements observed. The physical exercise or multicomponent exercise programs allow for the establishment of a social relationship between the supervisors and the residents, on the one hand, and between the residents themselves, on the other. This social relationship is an important factor that can affect their quality of life (Barthalos et al., 2016).

### Explanations for the Inefficiency of Physical Exercise or Multicomponent Exercise Programs

Another finding of our review is that in older adults with dementia, several studies failed to show any beneficial effects of these interventions on mental health or cognitive functions. The lack of these effects could be explained by dementia-related factors, such as the disease progression during the intervention and fluctuations in cognitive functioning (Sanders et al., 2020). Another factor might be the low adherence of the residents to these interventions (Henskens et al., 2018). Therefore, it is important to develop strategies for older adults to engage effectively in physical exercise while residing in nursing homes. For this purpose, we could, for example, make use of the giant exercise board game interventions recently developed by a Belgian research team (Mouton et al., 2017; Buckinx et al., 2020) or exergames (Ismail et al., 2022).

### Requirements for Standardizing the Methods Used in the Studies of Older Adults Living in Nursing Homes

We also found a difference between the methods used by each study. The first difference was observed in the measurement of mental health. Different questionnaires were used to obtain indices of depression, anxiety and wellbeing. The second difference lied within the multicomponent interventions. In these interventions, different activities were added under physical exercise (readings, discussions on aging, skill administered acupressure and massage, depression awareness training and motor-cognitive games) depending on the study. The final difference was not only the great variability in the total duration of the interventions, which ranged from 8 to 52 weeks, but also the absence of an objective measurement of intensity in many studies. In this regard, it would be interesting to reflect on the standardization of the methodology used in studies that are centered around a topic such as this.

### Limitations and Future Research

Our systematic review remains one of the rare reviews to synthesize the effects of physical exercise or multicomponent exercise programs on the mental health and cognitive functions of older adults, regardless of whether they have dementia or use wheelchair assistance. However, it has some limitations, such as the inclusion of only three databases from which, only articles written in English were selected. This might have led us to omit key studies. In addition, we have not used other synonyms for “elderly,” which may lead to the omission of some studies. However, the fact of associating “elderly” with 'nursing homes' allowed us to find almost all the studies carried out in nursing homes where the residents are necessarily older adults. Another limitation is that four of the studies included in our systematic review have a poor methodology which could lead to biased interpretations in our review.

Future studies must examine the effects of an innovative and adaptive physical activity program on the mental health and cognitive functions of older adults living in nursing homes. These studies must go beyond the measurement of mental health and cognitive performance and look at the evolution of other variables such as C-reactive proteins. C-reactive proteins are a marker of inflammation (Fedewa et al., 2017) that, once measured, will allow us to verify the inflammatory hypothesis in older adults by establishing a correlation between the protein and cognitive performance. Another avenue of research that is scarcely investigated in this population is the influence of body image on wellbeing. In the literature, positive body image is correlated with body esteem which is an indicator of psychological wellbeing (Andrew et al., 2016). Thus, a physical exercise program to improve the body image and, therefore, the wellbeing of older adults living in nursing homes must be considered.

## Conclusion

This systematic review highlighted the effects of physical exercise or multicomponent exercise programs on the mental health and cognitive functions in older adults living in nursing homes. Specifically, these effects were more observed in older adults without dementia. However, the methods used in these studies need to be more standardized to provide additional reliable data for beneficial effects. In the future, it would be judicious to think about innovative interventions for helping older adults adhere better to interventions. It would also be fruitful to investigate the neurophysiological adaptations underlying the effects of these interventions on mental health and cognitive functions.

## Data Availability Statement

The original contributions presented in the study are included in the article/Supplementary Material, further inquiries can be directed to the corresponding author/s.

## Author Contributions

JD and NA contributed to conception and design of the review, selected and read the studies included in the review, organized the database on the systematic review, and have written the first draft of the manuscript. JD, NA, and CL wrote sections of the manuscript. All authors contributed to manuscript revision, read, and approved the submitted version.

## Funding

This work was supported by the Conventions Industrielles de Formation par la Recherche No. 2020/1236.

## Conflict of Interest

The authors declare that the research was conducted in the absence of any commercial or financial relationships that could be construed as a potential conflict of interest.

## Publisher's Note

All claims expressed in this article are solely those of the authors and do not necessarily represent those of their affiliated organizations, or those of the publisher, the editors and the reviewers. Any product that may be evaluated in this article, or claim that may be made by its manufacturer, is not guaranteed or endorsed by the publisher.
