# Supplementary material for: Effects of Chronic Physical Exercise or Multicomponent Exercise Programs on the Mental Health and Cognition of Older Adults Living in a Nursing Home: A Systematic Review of Studies From the Past 10 Years
Source: Front Psychol. 2022 May 13;13:888851. doi: 10.3389/fpsyg.2022.888851 (PMC9136454; doi:10.3389/fpsyg.2022.888851)
Supplement: Supplementary file 1 [file Table_1.pdf]

Supplementary Material Table 1. Studies conducted in older adults without dementia who live in a nursing home and do not require wheelchair assistance

References	Participants, Measures of interest variables)	Intervention	Summary of majors' findings
Arrieta et al. (2020)	**Experimental group**: N = 57 (42 women) ; Age: 85.1±7.6 years ; **Control group**: N = 55 (37 women) ; Age : 84.7±6.1 years ; **Well-being**: Quality of life in Alzheimer's Disease rating scale; **Anxiety and depression**: Goldberg anxiety and depression Scale ; **Cognitive functions**: MOCA (global cognition) ; Rey Auditory-Verbal Learning Test (verbal memory and capacity to recall and accumulate words through learning trials), Trail making test (executive functions), Coding and symbol search test (processing speed), Verbal fluency (cognitive functioning).	**Intervention**: Multicomponent physical exercise (strength, balance, walking). Participants attend two sessions per week for 6 months and each session lasted one hour. The intensity of the strengthening exercises ranged from 40% at first to 70% 1-RM. As regards walking, the goal was to walk every day for 20 minutes.<br>**Control**: Memory workshops, reading, singing, and other similar activities.	After six months, multicomponent exercise significantly improves global cognition (MOCA) and processing speed (symbol search test).<br><br>Quality of life of participants in the intervention group increased trend-wise (p = 0.051).
Barthalos et al. (2016)	**Experimental group 1, 2**: N = 11 – 11 (N.A women); Age: 79.64 – 75.35±7.96 – 11.91 years; **Control group**: N = 11 (N.A women); Age: 76.51±1.47 years ; **Well-being**: World health organization's quality of life questionnaire ; **Anxiety and depression**: Unmeasured ; **Cognitive functions**: Unmeasured.	**Intervention 1**: Resistance training twice a week for 45 min for 15 weeks<br>**Intervention 2**: Resistance training + Mental training (lectures, discussions on aging and quality of life).<br>**Control group**: No resistance training or mental training	While both experimental groups improved their social participation, there was no significant difference between the groups in quality of life.
Bischoff et al. (2021)	**Experimental group**: N = 14 (12 women); Age: 83.6±7.3 years; **Control group**: N = 10 (9 women); Age: 83.8± 5.7 years ; **Well-being**: Short form of the health survey, satisfaction with life scale ; **Anxiety and depression**: Unmeasured ; **Cognitive functions**: Unmeasured.	**Intervention**: Multicomponent training (balance, coordination, cognitive exercises, aerobic exercises, strength exercises, relaxing and stretching exercises). The exercise program consisted of 32 sessions for a period of 16 weeks. One training session lasted 45-60 min and took place twice a week.<br>**Control group**: Waiting-list control group	A multicomponent training provides a non-significant increase in life satisfaction.

Supplementary Material Table 1. (continued)

References	Participants, Measures of interest variables	Intervention	Summary of majors' findings
Fakhari (2017)	**Experimental group**: N = 27 (13 women); Age: 69.19±5.48 years ; **Control group**: N = 29 (17 women) ; Age : 69.34±5.03 years ; **Well-being**: Unmeasured ; **Anxiety and depression**: Beck Depression Inventory ; **Cognitive functions**: unmeasured.	**Intervention**: Participants attend Tai chi exercise three times per week for twelve weeks. Each Tai Chi exercise session lasted 20-25 min. **Control group**: Participants performed activities of daily living.	After twelve weeks, participants in experimental group significantly decreased their mean depression score
Frändin et al. (2016)	**Experimental group**: N = 129 (91 women) ; Age: 85±7.93 years ; **Control group**: N = 112 (88 women) ; Age : 84.5±7.3 years ; **Well-being**: Philadelphia Geriatric Center Morale Scale; **Anxiety and depression**: unmeasured ; **Cognitive functions**: Mini-Mental State Examination (global cognition), cognitive subscale of Functional Independence Measure (comprehension, expression, social interaction, problem solving).	**Intervention**: Ordinary care and treatment + Physical and daily activities (walking or rising from a chair, strength, or balance. Participants completed 10 to 13 weeks of intervention overall at a rate of 93 min per week. **Control group**: ordinary care and treatment.	At the end of the intervention, no significant improvement was observed. On the contrary, the social and cognitive functions of the participants who followed the intervention deteriorated.
Kim & Kang (2021)	**Experimental group**: N = 20 (14 women) ; Age: 80.6±8.1 years ; **Control group**: N = 20 (17 women) ; Age : 82.6±9.3 years ; **Well-being**: Life satisfaction scale ; **Anxiety and depression**: Geriatric anxiety inventory & Geriatric depression scale ; **Cognitive functions**: Mini-Mental State Examination (global cognition).	**Intervention**: Regular activity therapy + Korean Trot music including rhythmic exercises such as hand clapping, stretching, dancing, or percussion. The intervention implemented biweekly for 12 weeks. Each intervention lasted 50 min. **Control group**: Regular activity therapy (recreation, physical exercise and/or counselling).	Music intervention with rhythmic exercises improved global cognition, depression, anxiety, and life satisfaction.

Supplementary Material Table 1. (continued)

References	Participants, Measures of interest variables	Intervention	Summary of majors' findings
Lok et al. (2017)	**Experimental group**: N = 40 (17 women) ; Age: > 65 years; **Control group**: N = 40 (19 women) ; Age: > 65 years; **Well-being**: SF-36 Quality of life questionnaire ; **Anxiety and depression**: Unmeasured & Beck depression inventory ; **Cognitive functions**: Unmeasured.	**Intervention**: Rhythmic exercises (40 min including 10 min warm-up and 10 min cool-down) + free walking (30 min). Participants attend the intervention four times per week for 10 weeks.<br>**Control group**: No planned implementation.	Individuals of the intervention group presented a significant decrease depression after the intervention compared to control participants.<br>Quality of life improved only in the intervention group.
Moreira et al. (2018)	**Experimental group**: N = 24 (24 women); Age: 84.83±4.11 years ; **Control group**: N = 21 (21 women) ; Age : 82.3±3.23 years ; **Well-being**: Unmeasured ; **Anxiety and depression**: Unmeasured ; **Cognitive functions**: MOCA.	**Intervention**: Intervention protocol was performed 3 times per week (50 min per session) for 16 weeks in multisensory exercise. Multisensory exercise consisted of the following blocks: warm-up, strength, coordination and balance, multisensory stimulation, and flexibility and cool-down.<br>**Control group**: nontreatment	Multisensory exercise program showed statistically significant improvements on cognition.
Rezola-Pardo et al. (2019)	**Experimental group**: N = 43 (28 women); Age: 85.3±7.1 years ; **Control group**: N = 42 (29 women) ; Age : 84.9±6.7 years ; **Well-being**: Quality of life Alzheimer's disease ; **Anxiety and depression**: Anxiety and depression Goldberg Scale ; **Cognitive functions**: MOCA (global cognition), Symbol search and coding tests from Wechsler Adult Intelligence Scale (processing speed), Semantic fluency test, Verbal fluency test , Rey Auditory Verbal Learning Test (verbal memory), Trail Making test (psychomotor speed).	**Intervention**: Multicomponent program consists of to perform two sessions (strength, balance stretching exercises and walking) per week lasting approximately an hour each. The intensity of the strengthening exercises ranged from 40% at first to 70% 1-RM. Multicomponent program lasted 3 months.<br>**Control group**: Multicomponent program + dual-task training (walk + cognitive task).	Both interventions were effective in maintaining cognitive function. Only the multicomponent group significantly reduced anxiety.<br>Only the multicomponent group significantly improved quality of life.

Supplementary Material Table 1. (continued)

References	Participants, Measures of interest variables	Intervention	Summary of majors' findings
Rezola-Pardo et al. (2020)	**Experimental group**: N = 41 (26 women); Age: 84.7±6.5 years ; **Control group**: N = 40 (27 women) ; Age: 83.8±6.2 years ; **Well-being**: Quality of life Alzheimer's disease ; **Anxiety and depression**: Anxiety and depression Goldberg Scale ; **Cognitive functions**: MOCA (global cognition), Rey Auditory Verbal Learning Test (verbal memory).	**Intervention**: Multicomponent program consists of to perform two non-consecutive sessions (strength, balance stretching exercises and walking) per week lasting approximately an hour each. The intensity of the strengthening exercises ranged from 40% at first to 70% 1-RM. Multicomponent program lasted 3 months. **Control group**: Walking. The goal is to gradually increase the walking time to 150 min per week.	No significant differences were observed in cognitive performance. Both groups showed improvements in anxiety and quality of life. Only walking group significantly diminished depression score.
Tapps et al. (2013)	**Experimental group**: N = 20 (14 women); Age: N.A ; **Control group**: N = 20 (14 women) ; Age : N.A ; **Well-being**: Unmeasured; **Anxiety and depression**: Unmeasured & Beck depression Inventory ; **Cognitive functions**: Unmeasured.	**Intervention**: Participants were asked to participate in 12-week resistance based physical activity. Participants performed the exercises three times per week at 30 min per session. **Control group**: Activities provided by the LTC facility.	Results suggested that resistance based physical activity has the potential to lower perceived depression scores.
Tse et al. (2014)	**Experimental group**: N = 225 (182 women); Age: 85.45±6.25 years; **Control group**: N = 171 (135 women); Age: 85.44 ± 6.35 years ; **Well-being**: Health survey short form questionnaire, Life satisfaction index ; **Anxiety and depression**: Unmeasured & Geriatric depression scale ; **Cognitive functions**: Unmeasured.	**Intervention**: Physical exercise program (strengthening, stretching, balancing, towel dance, skill administered acupressure and message). The intervention was delivered one time a week for eight weeks. Each session lasted for an hour. **Control group**: No treatment during the study period.	Life satisfaction and depression of the experimental group was significantly improved.

Supplementary Material Table 1. (end)

References	Participants, Measures of interest variables	Intervention	Summary of majors' findings
Underwood et al. (2013) *	**Experimental group**: N = 398 (294 women) ; Age: 86.7±7.2 years ; **Control group**: N = 493 (383women) ; Age: 86.3±7.5 years ; **Well-being**: European quality of life-5 ; **Anxiety and depression**: unmeasured and geriatric depression scale-15 ; **Cognitive functions**: Mini-mental state examination (global cognition).	**Interventional**: Depression awareness training + twice-weekly physiotherapist-led 45 min sessions ran for up to 12 months. The exercise sessions were designed to provide a moderate intensity strength and aerobic training stimulus + encouragement to do physical activity in daily life. **Control group**: Depression awareness training.	Intervention did not reduce depressive symptoms in residents of care homes. No evidence of a difference between intervention and control groups on well-being.

N = Number of attendees; NA = Not available; MOCA= Montreal Cognitive Assessment; 1-RM = One repetition maximum

*: The sample in the underwood et al. study contained 29% of older adults with dementia. Because the percentage of older adults with dementia is significantly lower than the percentage of older adults without dementia, we classified this study as work that included people without dementia.

Supplementary Material Table 2. Studies conducted in older adults with dementia who live in a nursing home or do require wheelchair assistance

References	Participants, Measures of interest variables	Intervention	Summary of majors' findings
Chen et al. (2015)	**Experimental group**: N = 59 ; **Control group**: N = 55 ; Mean age of all participants was 79±7.3 years ; Women represented 49.12% of the total population ; **Well-being**: Unmeasured ; **Anxiety and depression**: Unmeasured & Taiwanese Depression Questionnaire ; **Cognitive functions**: Unmeasured.	**Intervention**: Regular daily activities + Wheelchair-bound senior elastic band (WSEB) exercise program. The WSEB group exercise intervention was performed three times per week 40 min per session for 6 months.<br>**Control group**: Regular daily activities.	Participants in the intervention group had less depression than the control group at 3 months of the study and maintained them throughout the rest of the 6-month study.
Cheng et al. (2014)	**Experimental group**: N = 39 (64% women); Age: 81.8±7.4 years ; **Control groups**: N = 35, 36 (66, 64% women) ; Age : 80.9±7.2 years, 81.9±6.2 years ; **Well-being**: Unmeasured ; **Anxiety and depression**: Unmeasured and Geriatric depression scale ; **Cognitive functions**: Mini-mental State Examination (global cognition), Forward and backward digit sequence, 15-word immediate and 30-minute delayed recall, categorial verbal fluency (animals, fruits, and vegetables), Impaired word-list delayed recall (tapping episodic memory) and categorical fluency (semantic memory).	**Intervention**: Tai chi<br>**Control group 1**: Mahjong (Cognitive stimulation)<br>**Control group 2**: Handicrafts.<br>In each group, participants practiced the activities for one hour, three times a week for 12 consecutive weeks.	In contrast to the participants in control group 2, the participants who followed the Tai Chi program or the cognitive stimulation showed an improvement in global cognition and memory (delayed recall and/or forward digit span).
Cordes et al. (2021)	**Experimental group**: N = 26 (65% women); Age: 82.69±10.26 years ; **Control group**: N = 26 (62% women) ; Age : 80.12±11.37 years ; **Well-being**: 2 items of short form of the Health Survey SF-12 ; **Anxiety and depression**: Unmeasured & Center for epidemiological studies depression Scale (CES-D); **Cognitive functions**: MOCA (global cognition), serial subtraction test (working memory).	**Intervention**: Multicomponent chair-based exercise program (coordination, motor-cognitive games, task-specific exercises, strength, aerobic and stretching exercises). Exercise program consisted of 32 sessions and was conducted in a period of 16 weeks. One training session lasted 60 min and training took place twice a week.<br>**Control group**: Regular everyday activities.	Participants of multicomponent chair-based exercise program improve cognitive functions (global cognition and working memory) and depression.

Supplementary Material Table 2. (continued)

References	Participants, Measures of interest variables)	Intervention	Summary of majors' findings
de Souto Barreto et al. (2018)	**Experimental group**: N = 44 (41 women); Age: 88.3± 5.1 years; **Control group**: N = 47 (36 women); Age: 86.9±5.8 years ; **Well-being**: Unmeasured ; **Anxiety and depression**: Unmeasured ; **Cognitive functions**: Mini-mental state examination.	**Intervention**: Multicomponent (coordination and balance exercises, strengthening, aerobic exercise. The program took place twice per week for 60 min per session for 24 weeks. Exercise<br>**Control group**: Social activity (therapeutic music mediation or arts and crafts).	No effect of the intervention on global cognition.
Henskens et al. (2018)	**Experimental group 1, 2, 3**: N = 21 - 22 - 22 (90,5-77,3 - 63,4 % women); Age: 86.05 – 85.14 – 86.95± 5.86 - 4.64 - 7.21 years ; **Control group**: N = 22 (77,3% women) ; Age : 84.73±4.55 years ; **Well-being**: Unmeasured ; **Anxiety and depression**: Unmeasured & Cornell Scale for depression ; **Cognitive functions**: Mini-mental state examination (global cognition), Severe impairment battery-Short Form, category fluency subtest of the Groninger Intelligence Test, Wechsler digit span task backward, go-no-go (executive functions), Digit span task forward (attention).	**Intervention 1**: Activities of daily living **Intervention 2**: Exercise training (aerobic and strength) - **Intervention 3**: Combined exercise and activities of daily living. Exercise sessions took place 3 times per week for 6 months. Each lasted 30-45 min.<br>**Control group**: Participants drank tea with the nursing staff 3 times per week.	- 6-month activities of daily living benefitted executive functions and depression among men.<br><br>- Exercise training has no effect on cognitive function or depression.<br><br>- Combined training benefitted depressive symptoms compared to exercise training.
Sanders et al. (2020)	**Experimental group**: N = 39 (53,8% women); Age: 81.7±7.16 years ; **Control group**: N = 30 (73,3% women) ; Age : 82.1±7.51 years ; **Well-being**: Unmeasured ; **Anxiety and depression**: Unmeasured ; **Cognitive functions**: Mini-mental state examination (global cognition), TMT A (psychomotor speed), Digit Span Forward and backward (verbal memory span and visual working memory), Stroop test (attention and inhibitory control), Phonemic fluency test (executive function).	**Intervention**: Aerobic sessions (12 weeks of low intensity, 57-63% heart rate maximal and 12 weeks of high intensity, 83-89% heart rate maximal) + Lower limb strength exercises (12 weeks low intensity, 9-11 RPE and 12 weeks high intensity 13-16 RPE).<br>**Control group**: Flexibility exercises and recreational activities	There were no significant effects of exercise vs. control intervention on any cognitive measures.

Supplementary Material Table 2. (end)

References	Participants, Measures of interest variables	Intervention	Summary of majors' findings
Telenius et al. (2015a)	**Experimental group**: N = 82 (72 % women); Age: 86.9±7 years; **Control group**: N = 81 (75,3 % women); Age: 86.4±7.8 years ; **Well-being**: Quality of life in late-stage dementia scale ; **Anxiety and depression**: Unmeasured & Cornell Scale for depression in Dementia ; **Cognitive functions**: Mini-mental state examination (global cognition).	**Intervention**: High intensity functional exercises. Exercise sessions lasted 50-60 min (strengthening exercises, balance exercises). Participants exercised twice a week for 12 weeks. Intensity of strengthening exercises aimed to be 12 repetitions maximum. **Control group**: Participants met in groups twice a week for 50-60 min control activities (light physical activity, reading, playing games, listening to music and conversations.)	No significant improvement was found at the end of the program for participants in the experimental group on cognitive variables and quality of life.
Telenius et al. (2015b)	**Experimental group**: N = 87 (63% women); Age: 87.3±7.0 years ; **Control group**: N = 83 (62% women) ; Age : 86.5±7.7 years ; **Well-being**: Quality of life in late-stage dementia scale; **Anxiety and depression**: Unmeasured & Cornell Scale for depression in Dementia ; **Cognitive functions**: Mini-mental state examination (global cognition).	**Intervention**: High intensity functional exercises. Exercise sessions lasted 50-60 min (strengthening exercises, balance exercises). Participants exercised twice a week for 12 weeks. Intensity of strengthening exercises aimed to be 12 repetitions maximum **Control group**: Participants met in groups twice a week for 50-60 min control activities (mobility exercises and stretches, reading, playing games, listening to music, and making conversations).	No significant improvement was found at the end of the program for participants in the experimental group on cognitive variables and quality of life.

MOCA= Montreal Cognitive Assessment; RPE = Rate of perceived exertion
